# Families Affected by Parental Cancer: Quality of Life, Impact on Children and Psychosocial Care Needs

**DOI:** 10.3389/fpsyt.2021.765327

**Published:** 2021-11-10

**Authors:** Laura Inhestern, Lene Marie Johannsen, Corinna Bergelt

**Affiliations:** ^1^Department of Medical Psychology, University Medical Center Hamburg-Eppendorf, Hamburg, Germany; ^2^Department of Medical Psychology, University Medicine Greifswald, Greifswald, Germany

**Keywords:** family, cancer, oncology, quality of life, psychosocial needs

## Abstract

Parental cancer poses major challenges for families with minor children. Due to diagnosis and treatment family life is disrupted. To prevent long-term consequences in all family members and to design needs-oriented family-centered interventions, further understanding of the family's situation including the impact on the children, quality of life levels and the parental psychosocial needs is necessary. This study aims at investigating the impact of parental cancer on the minor children, family-specific psychosocial needs and quality of life levels of parents and children. Cancer patients parenting at least 1 minor child (<18 years) were eligible for study participation. In total, n=86 cancer patients under treatment participated in the study. After excluding participants without a minor child, 78 patients remained for analyses. We assessed children's quality of life using the parent proxy version of the KIDSCREEN-10 and parental quality of life using the EORTC QLQ C30 quality of life questionnaire. Additionally, the questionnaire comprised open questions about positive and negative changes parents perceived in their children as well as questions on specific family- and child-related psychosocial needs. The majority of participants were mothers (91%), mainly diagnosed with breast cancer (59%). The participating parents provided data on 117 minor children. Parents mentioned positive changes in 38% of the children (e.g., being more attentive and helpful). Negative changes were reported in 37% of the children (e.g., being more anxious and clingy). Parents reported family-specific psychosocial supportive care needs for themselves as a parent (e.g., support regarding parenting concerns), support needs for the partner or the children. Moreover, parents expressed family-related information needs and needs regarding practical aspects (e.g., childcare, household help). Global quality of life was *M* = 55.7 (*SD* = 23.4) for parents and *M* = 57.5 (*SD* = 15.5) for children. Pearson's correlation coefficient between parental and children's quality of life was 0.377 (*p* < 0.001). To identify parents with cancer and children in need for additional support, health care providers should proactively inquire about the impact of the disease on the children. In terms of a comprehensive cancer care, the direct assessment of family impact and family-specific support needs in cancer patients with minor children allows for needs-based allocation to support offers.

## Introduction

Living with cancer imposes mental and physical challenges on the patients and demands adjustment processes from the patients but also their relatives. A cancer diagnosis in rather young adulthood can meet the patient in a phase of enhanced responsibility for the family (1). About 14% of cancer patients live with minor children (2).

Most children adjust well to the parental disease without developing relevant psychopathological symptoms (3, 4). Still, they are confronted with cancer-related stressors (5, 6) and can experience elevated stress levels and subclinical levels of increased mental burden (3, 7). Studies indicate that besides disease-related factors (e.g., progress or side effects), parental coping style, family functioning and specifically open communication influence children's well-being and adjustment (8). As these aspects can be addressed in psychosocial interventions, the systematic assessment of support needs of affected parents is relevant to identify patients in need for support and may raise the awareness of these aspects in health care personnel. Initiating needs-based support may prevent negative long-term consequences in children, e.g., by promoting open family communication. So far, predominantly qualitative studies have identified specific needs of affected parents and their families (9, 10). Studies demonstrated that parents are concerned about the emotional well-being of their children (11, 12). Moreover, cancer patients experience parenting demands, while caring for their own needs as patients (13). Parents report several support needs e.g., in communicating with their children about the disease and in obtaining a professional assessment of children's reaction to cancer (11). While supportive and psychosocial care needs are a common parameter in studies on cancer patients in general, the specific needs of affected parents were only rarely investigated to date (14, 15). In a recent study on breast cancer patients, affected mothers report a high level of needs for themselves as well as for their children (15).

Quality of life in children and parents affected by parental cancer have previously been investigated. Parents with cancer report lower health-related quality of life than population-based norm values (16). Interestingly, quality of life in minor children of cancer patients is rather scarcely investigated compared to the psychological impact such as internalizing and externalizing problems (17–19). Several factors associated with parental quality of life have been identified in previous studies, e.g., gender, partnership status, or time since diagnosis (20, 21). Moreover, higher depressive symptoms in parents with cancer are associated with higher parenting stress (22) and hence may impact children's well-being (23). With regard to children's quality of life, family functioning, palliative treatment, and family-related factors such as parental gender or single parenthood have been identified as associated factors (16, 24).

Besides quality of life, distress or psychopathology, the direct assessment of the impact of the parent's disease on the children can provide crucial information regarding the need for psychosocial support in the children. Therefore, the research questions of this study are:

1. How do parents with cancer perceive the impact of their illness on their minor children?
2. How frequent do affected parents report family-specific psychosocial needs?
3. How are quality of life levels in family members when a parent has cancer and how are they related to each other?

## Methods

We used baseline data from a longitudinal quantitative study with two measurement points (baseline, 6 months follow up).

### Data Collection and Participants

The study was conducted at a major cancer center in Hamburg, Germany, providing inpatient and outpatient treatment as well as psychooncological routine care and additional child-oriented support service, if applied for. Cancer patients, receiving any kind of treatment (inpatient, outpatient, medical, psychooncological) were invited for participation.

Since a systematic assessment of parenthood status was missing, potentially eligible patients were addressed either based on their age (<55 years) or based on information from clinical staff. Patients parenting at least one minor child (<18 years) were given study information and invited for participation. If they agreed, they received the set of questionnaires, written study information and informed consent as well as an addressed envelope to send study documents to the study center. Data was collected between January 2016 and August 2018.

**Figure 1:**
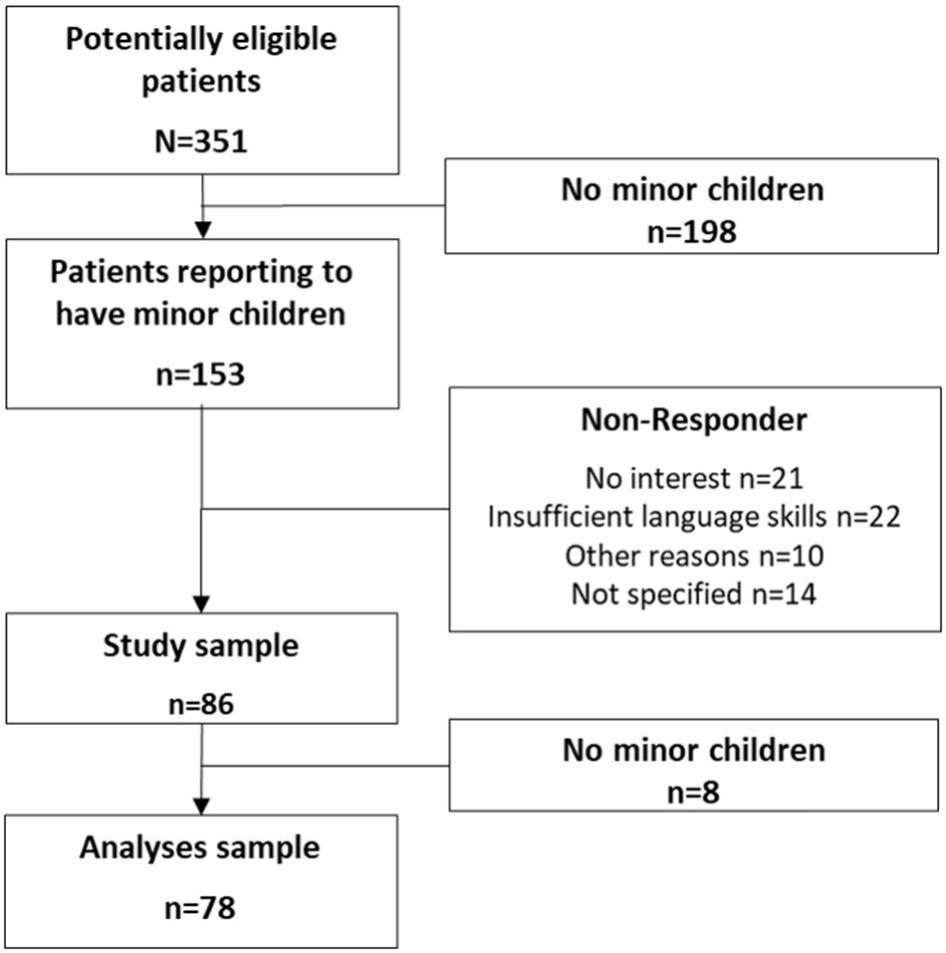
Flow chart of data collection.

Three hundred and fifty one patients aged between 18 and 55 years were informed about the study. Of these, 153 stated to be parents of at least one minor child and were thus invited to participate in the study. Eighty six patients filled out the questionnaire and returned it. After excluding the participants without a minor child, 78 patients were included in the analyses (Figure 1).

### Measures

Sociodemographic and disease-related information was obtained by self-report questions.

The impact of the parental disease on the children was assessed by asking parents if (1) positive and (2) negative changes in their children occurred associated to parental cancer (binary response format for each question: yes/no). If parents answered “yes,” they were asked to explain the changes in an open response format. Moreover, parents could answer “no changes associated to cancer.” Open answers were analyzed by qualitative content analysis (25).

Psychosocial supportive care needs were assessed using a self-developed set of 25 items that were developed based on results of previous studies of the research group (26). Patients were asked to rate 10 psychosocial support needs for themselves as a parent, 4 needs for their children, 3 needs for the partner (if applicable), 3 needs for the family as a whole and 5 items on organizational and practical needs as a parent. Moreover, one open question on further needs was included. Patients could agree or disagree on each item.

Parental quality of life was assessed using the global scale and the function scales of the European Organization for Research and Treatment of Cancer Quality of Life Questionnaire (EORTC QLQ-C30) (27). The EORTC QLQ-C30 comprises multiple dimensions of health-related quality of life including five function scales, nine symptom scales and one global quality of life scale. The thirty items can be rated on a 4-point Likert scale (1 = *not at all* to 4 = *very much*). The items of the scales are summed up and transformed into a scale from 0 to 100 with higher values indicating better quality of life in the function scales and the global scale. The EORTC QLQ-C30 is widely used and has proven to be reliable and valid (28).

To assess the children's quality of life, we used the KIDSCREEN 10 Index proxy version (29). Cancer patients answered the 10 items of the KIDSCREEN on a 5-point Likert scale (1 = *not at all* to 5 = *extremely*). The original version is recommended for children from 8 to 18 years, since items on school performance are included and do not apply for younger children.

Our study also included patients with children younger than 8 years. However, due to study organization, we could only apply one instrument for all patients. In case of one missing item, missing data was replaced using the parameter values from the remaining items. If more than one item was missing, the case was coded as missing. Consequently, we replaced missing, not applicable items in children younger than 8 years with the rounded mean raw item score of the remaining items. As recommended by the original authors, we transformed the scores into *T*-values (*M* = 50, *SD* = 10). Higher values indicate higher quality of life. The KIDSCREEN 10 parent proxy report (version for children between 8 and 18 years) has shown good psychometric properties with good internal consistency (Cronbach's Alpha = 0.78) and good test-retest reliability (*r* = 0.67) (30).

### Data Analyses

Statistical analyses were conducted using the statistical package for the social sciences (SPSS, version 27, IBM SPSS, Chicago). Descriptive statistics were computed for sociodemographic, disease-related and study outcomes. Pearson's correlation coefficient was calculated to assess the association between parental and child's quality of life. To avoid inaccurate results due to same family affiliation of some children, we randomly selected one child per patient for the correlation analysis. The significance level was p=0.05 for all analyses.

### Ethics

The study was approved by the local Ethics Committee for Psychotherapists (18/2014-PTK-HH, 15/2015-PTK-HH).

## Results

### Sample Characteristics

The sample comprises n=78 cancer patients parenting minor children, mainly comprising women (*n* = 71). Patient's age ranged from 29 to 53 years (*M* = 42.1, *SD* = 5.9). Most participants had one (48%) or two children (34%). 36% reported school education ≤ 10 years; 64% of participants were employed in full- or parttime. Participants were mostly diagnosed within the year before the survey (75%), mainly with breast cancer (62%). About one quarter of the participants had used family- or child-related psychosocial support at least once (Table 1).

**Table 1:** Sample characteristics of *n* = 78 cancer patients parenting minor children.

**Variable**	***n***	**%**
Female	71	91.0
Age (M, SD)	42.2 (5.9)	
**School education**
≤ 10 years	28	35.9
11–13 years	50	64.1
Living in a partnership	68	87.2
**Number of children**
1	38	48.7
2	27	34.6
3	12	15.4
≥4	1	1.3
Currently on sick leave	48	61.5
Employed in full- or part time	50	64.1
**Time since diagnosis**
≤ 1 year	58	75.3
>1 year	19	24.7
Missing data	1	1.3
**Treatment**
Surgery	54	69.2
Chemotherapy	77	98.7
Radiation	28	35.9
**Tumor diagnosis**
Breast	49	62.8
Gynecological	14	18.7
Other	12	15.4
Missing data	3	3.8
Use of family- or child-related psychosocial support	20	25.6

Overall, participants reported on 117 minor children between 0 and 17 years (*M* = 8.4, *SD* = 4.9). 52% of the children were daughters and 47% sons (4 missing values).

### Assessment of the Impact of Cancer on the Children

**Figure 2:**
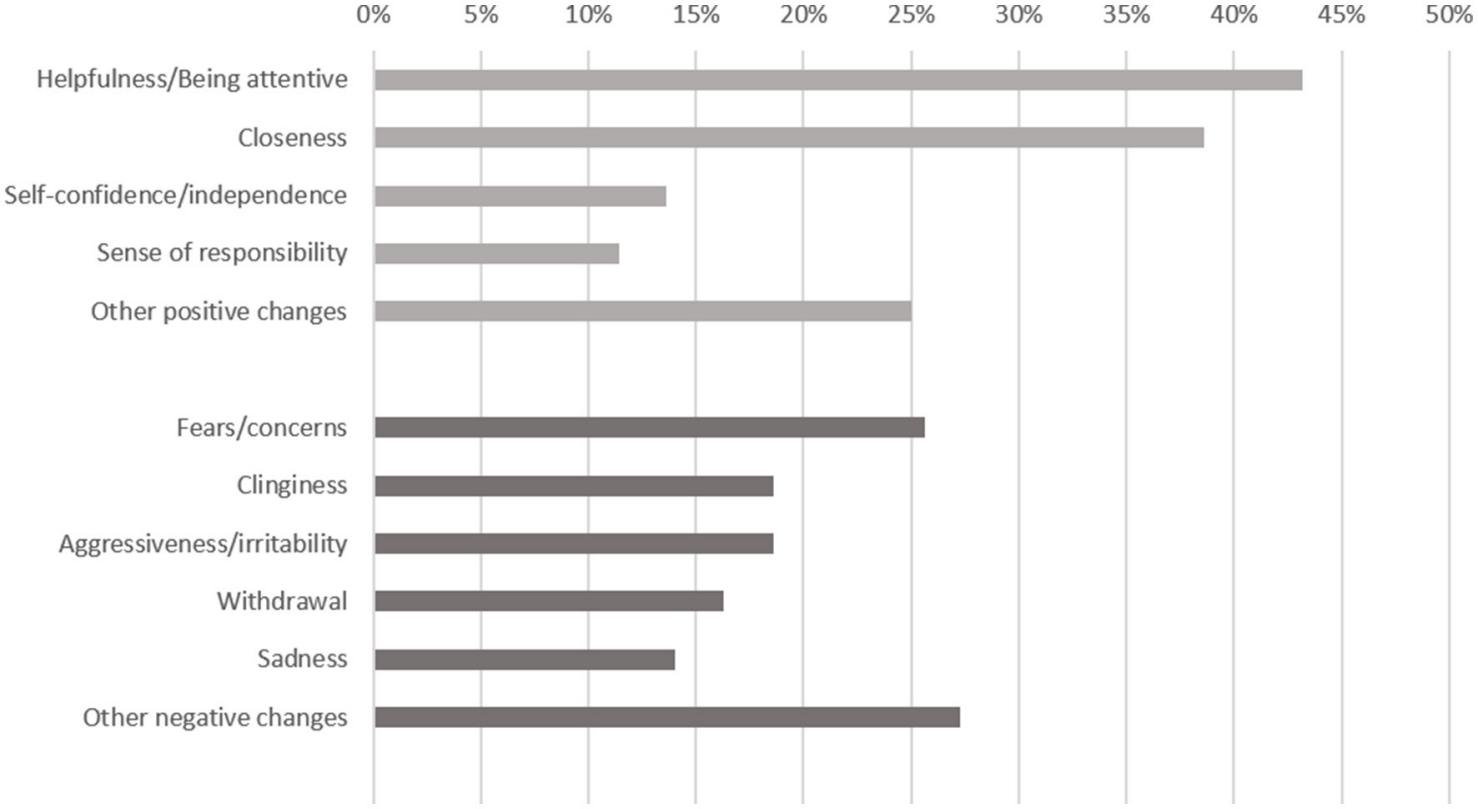
Positive (light gray) and negative (dark gray) changes in children affected by parental cancer (parent report, *n* = 44 children with positive changes, *n* = 43 children with negative changes).

Parents reported in 37% of the children both a positive and a negative impact of the parental cancer disease. All parents indicating changes in their children answered the open question with at least one change, which they had observed in their child. Most frequently reported positive changes were helpfulness (reported in 19 of 44 children) and more closeness within the family (17 of 44 children) (Figure 2). Other reported positive changes were increased self-confidence and sense of responsibility as well as better school performance or being more open in social situations. Most frequently reported negative changes were fears and concerns (11 of 43 children), increased clinginess and aggressiveness/irritability in the children (each 8 of 43 children). Moreover, parents reported higher levels of sadness, withdrawal, difficulties to concentrate or sleeping difficulties.

### Family-Specific Psychosocial Needs

About three out of four parents reported the need for information about children's coping (74%). Other frequent needs were support in dealing with the children, their feelings and their behavior (71%) and support regarding concerns as a parent (64%) (Table 2).

**Table 2:** Family-specific psychosocial needs, *n* = 78.

**Psychosocial need**	***n***	**%**
**Needs as parent**		
Psychological support for myself to be available for the needs of my children	40	51.3
Better information flow about my family status in the clinic and other health care institutions	29	27.2
Support to cope with feelings of guilt	26	33.3
Support regarding my concerns as a mother/father	50	64.1
Support in dealing with my children, their feelings and their behavior	55	70.5
Information about children's coping with a severe parental illness	58	74.4
Support in telling my children about the illness	49	62.8
Information about genetic predisposition and heredity	45	57.7
Professional assessment of the behavior and coping of my children	37	47.4
Exchange with other affected parents	23	29.5
**Perceived needs for children**		
Exchange with peers in similar situations	21	26.9
Support in coping with the parental illness	39	50.0
Support regarding school	16	20.5
Age-appropriate information about the illness	55	70.5
**Perceived needs for the co-parent**		
Support in dealing with our children, their feelings and their behavior	46	59.0
Exchange with other affected co-parents	19	24.4
Support in coping with the illness	44	56.4
**Family needs**		
Support in communicating about the illness	37	47.4
Exchange with other affected families	16	20.5
Activities/facilities for the whole family	51	65.4
**Organizational and practical needs**		
Support in childcare	38	48.7
Information on household help or other support offers	50	64.1
Information on financial support offers	43	55.1
Information on the topic “cancer and desire to have children”	6	7.7
General information on “parenthood and cancer”	45	57.7

Regarding their children, the parents assessed the need for age-appropriate information (71%) and support in coping with the parental illness (50%) as most frequent needs. Moreover, more than half of the patients reported support needs for their co-parent in coping with the illness (56%) and in dealing with the children, their feelings and behavior (59%).

Other frequent needs were “activities/facilities for the whole family” (65%) and organizational and practical needs, such as information on household help (64%), financial support (55%) or general information on the topic parenthood and cancer (58%).

Only two parents used the possibility to add another need and mentioned “financial consequences” and “information on supporting non-profit associations.”

### Quality of Life

Global health status/quality of life of parents was *M* = 55.7 (*SD* = 23.4) on a scale from 0 to 100. Highest functioning was reported in the subscale physical function (M = 75.0, SD = 20.9), lowest functioning in the social function subscale (*M* = 40.7, *SD* = 32.7) (Table 3). All values were lower than population-based norms.

**Table 3:** Quality of life levels in parents with cancer and their children.

**Quality of life**	***n***	***M***	***SD***	**Norm values^c, d^**
**Parental quality** **of life^a^**				
Global health status/Qol	78	55.7	23.4	67.0
Physical Function	59	75.0	20.9	82.8
Role Function	78	49.6	35.1	80.8
Emotional Function	77	49.0	26.2	73.9
Cognitive Function	78	63.9	28.5	83.9
Social Function	77	40.7	32.7	84.8
**Children's quality** **of life^b^**				
General health index	100	57.3	15.8	51.91 (8–11 year old children)49.00 (12–18 year old children)

a *EORTC QLQ-C30, Global functioning and functioning subscales, 0-100*.

b *KIDSCREEN 10 Index, proxy version, T-values*.

c *(31); EORTC QLQ-C30 general population norm data for Germany, weighted scores*.

d *(29), European norm data, proxy version*.

Children's general health index was rated *M* = 57.3 (*SD* = 15.8) on a T-transformed scale.

Pearson's correlation coefficient between parental and child's quality of life was 0.377 (*p* < 0.001, *n* = 74).

## Discussion

Results demonstrate that parents experience both positive and negative changes in more than one third of their children. Besides burden in their children, parents notice elements of posttraumatic growth as it has been identified in adult cancer patients (32). This indicates that open questions on changes in children allow for a deeper insight into the specific impact of parental cancer on children rather than structured questionnaires using closed question format e.g., on quality of life or psychopathological symptoms. In particular, in the context of the clinical oncological setting, a combination of direct and indirect measurement of disease impact may be a promising approach to capture a valuable insight into the situation of children affected by parental cancer. Mostly, the parents are the key informants for their children's situation and gate keepers to use of psychosocial support services for their children. A consequent inclusion of the parental role of cancer patients with minor children and a proactive approach by clinicians in addressing these aspects are essential to provide preventive psychosocial support for the whole family (33, 34).

While a substantial number of studies have investigated supportive care needs in cancer patients, so far only few studies assessed the specific needs of families affected by parental cancer (15). The applied list of parental psychosocial needs seems to represent their needs adequately since there are high agreement rates and only few participants made use of the free-response format in case of additional needs. Similar to the findings of Hammersen and colleagues (15), our results show a high number of family- and parent-specific needs in affected parents. More than two thirds of parents wish information on how children cope with a parental disease and support on how to deal with the children and their feelings. Almost half of the parents wish for a professional assessment of the children's behavior, which indicates limited parental self-efficacy. Comparing self-reported parental self-efficacy before and after diagnosis, it declines significantly after the diagnosis (35) and parents become unsure whether they are still be able to meet their children's needs adequately. In a study on use and need of psychosocial support in cancer patients with minor children, it was identified, that 73% of the participants expressed the need for support, but only 9% used family-specific support (14). A more concise and structured assessment of family-specific needs in routine care might help the needs-based allocation of adequate psychosocial support offers. Besides support initiated by health care teams, families might benefit from support in daily life at home. Household help or social legal advice as well as children's books, brochures for affected families or organization of additional child care can be helpful for affected families (36). Moreover, children can be supported in daycare or school. Staff can provide support and information and may serve as additional person of trust outside the family setting (37, 38). In school, structures and routines are perceived as stabilizing factors for most children (37).

Children's level of quality of life was similar to other studies in children of cancer survivors (16). Since the mean age of children in our study was 8 years, we might not have included the highly burdened, vulnerable children of older age groups. Jeppesen and colleagues reported in a sample of adolescents that 42% participants had low quality of life levels (39). Moreover, adolescents themselves report high levels of unmet needs (40), which may not be captured by quality of life assessment. Similar to other studies (41), we found moderate positive correlations between parental and children's quality of life. However, studies on the impact of parental cancer on children using outcomes such as distress or mental health including internalizing and externalizing symptoms revealed that parents underestimated the impact of cancer on their children (3). Since we could not obtain child self-report, but could only include the parental perspective on family-specific needs and their children's quality of life in our study, there might be an underestimation of the children's actual needs and an overestimation of the quality-of-life levels, respectively. At the same time, the direct assessment of reactive changes due to parental cancer disease showed a high amount of positive and negative changes observed by the parents in our sample. This indicates that the participating parents are attentive towards their children and acknowledge both positive and negative impact of the situation. Still, parental report should be considered with caution, since own burden and less psychosocial availability of parents may lead to underestimation of children's burden.

### Limitations

There are several limitations to this study. First, the cross-sectional design does not allow to draw any causal relations between findings. A systematic longitudinal design across several milestones of cancer diagnosis and treatment is necessary in order to picture the course of changes, needs and quality of life. Moreover, the perspective of the children themselves and, hence, an important informant on children's needs and quality of life was not included. Third, results should be interpreted with caution due to the limited sample size and participation bias (mainly women and highly educated parents, high use of family-centered support). Lastly, the limited sample size did not allow for comprehensive analyses including multiple potential confounders.

## Conclusion

To identify parents with cancer and children in need for additional support, health care providers should ask openly and proactively about impact of the disease in the children. Due to limited time and personnel resources in the health care setting, needs-based allocation is essential in health care for cancer patients. The direct assessment of family-specific support needs in cancer patients with minor children allows for provision of specific support offers (e.g., psycho-oncologist, family-centered support offers, social work, social legal advice, information resources).

Since parental and children's quality of life show medium correlation, the assessment of the parental quality of life may not be sufficient to capture the situation of the whole family. Hence, in clinical practice, health care providers should assess the mental health and quality of life of all family members in order to identify those burdened.

## Data Availability Statement

The data that support the findings of this study are available from the corresponding author on reasonable request. The data are not publicly available due to privacy and ethical restrictions. Requests to access the datasets should be directed to Laura Inhestern, l.inhestern@uke.de.

## Ethics Statement

The studies involving human participants were reviewed and approved by Local Ethics Committee for Psychotherapists (18/2014-PTK-HH, 15/2015-PTK-HH). The patients/participants provided their written informed consent to participate in this study.

## Author Contributions

LI: study design and concept, data acquisition, statistical analysis and interpretation, and drafting of the manuscript. LJ: data acquisition and data interpretation and critical revision of the manuscript for important intellectual content. CB: study design and concept, data interpretation, and critical revision of the manuscript for important intellectual content. All authors approved the manuscript for publication.

## Funding

This project was part of a dissertation project funded by the Stiftung Wissenschaft in Hamburg, Germany. The funding source was not involved in study design, collection, analysis or interpretation of the data, in writing the manuscript or in the decision to submit the manuscript for publication.

## Conflict of Interest

The authors declare that the research was conducted in the absence of any commercial or financial relationships that could be construed as a potential conflict of interest.

## Publisher's Note

All claims expressed in this article are solely those of the authors and do not necessarily represent those of their affiliated organizations, or those of the publisher, the editors and the reviewers. Any product that may be evaluated in this article, or claim that may be made by its manufacturer, is not guaranteed or endorsed by the publisher.
